# Vitamin D status in children with a psychiatric diagnosis, autism spectrum disorders, or internalizing disorders

**DOI:** 10.3389/fpsyt.2022.958556

**Published:** 2022-09-14

**Authors:** Jet Muskens, Helen Klip, Janneke R. Zinkstok, Martine van Dongen-Boomsma, Wouter G. Staal

**Affiliations:** ^1^Karakter Child and Adolescent Psychiatry University Centre, Nijmegen, Netherlands; ^2^Department of Psychiatry, Radboud University Medical Centre, Nijmegen, Netherlands; ^3^Department of Psychiatry, University Medical Centre, Utrecht, Netherlands; ^4^Leiden Institute for Brain and Cognition, Leiden, Netherlands

**Keywords:** autism spectrum disorders, vitamin D, BMI, obesity, internalizing disorders, mood and anxiety disorders, child and adolescent psychiatry

## Abstract

**Background and objective:**

Multiple studies suggest that children with Autism Spectrum Disorders (ASD) have significantly lower vitamin D3 levels than typically developing children. However, whether vitamin D3 deficiency is more common in children with ASD than in children with other psychiatric disorders remains unclear. This study was conducted to explore the prevalence of vitamin D3 in children with a psychiatric diagnosis including children with ASD or with internalizing disorders (mood and anxiety disorders). In addition, this study investigated the potential associations between vitamin D3 and Body Mass Index (BMI).

**Materials and methods:**

Clinical data, including BMI and vitamin D3 levels, of 93 children (6–18 years; *n* = 47; 51% female) with ASD (*n* = 58) and internalizing disorders (*n* = 37) were retrospectively analyzed.

**Results:**

In the overall sample, the prevalence of vitamin D3 deficiency (<50 nmol/L) was 77.4%. Additionally, 75.9% of the children with ASD and 79.5% with internalizing disorders had vitamin D3 deficiency. BMI was inversely related to vitamin D3 in the total group (*p* = 0.016). The multiple regression model for the total group significantly predicted vitamin D3 (*p* = 0.022). Age contributed significantly to the prediction. Stratified for sex and primary diagnosis, multiple regression models showed that for boys with ASD, higher BMI levels were associated with lower vitamin D3 levels (*p* = 0.031); in boys with internalizing disorders and in girls, no relation was found between BMI and vitamin D3 levels.

**Conclusion:**

In this this cross-sectional, explorative study high rates of vitamin D3 deficiency in children with different psychiatric disorders were found. The results showed an inverse relation between BMI and vitamin D3 levels in the total group. Vitamin D3 deficiency was particularly common in boys with ASD and obesity. Lifestyle factors may contribute to the association between high BMI and low vitamin D3 levels in boys with ASD. Vitamin D3 deficiency is common in patients with psychiatric disorders and it is highly recommended to increase clinicians’ awareness of this common and remediable risk factor.

## Introduction

Autism spectrum disorder (ASD) is a neurodevelopmental disorder caused by a complex interaction between genetic and environmental risk factors (1–3). The combined data from several studies suggests an association between ASD and lower vitamin D3 status (4, 5). Vitamin D3 deficiency has been reported in a range of neuropsychiatric disorders across the lifespan, including for instance mood disorders, psychosis and different types of dementia (3–7). Reversely, in children with ASD lower vitamin D3 levels have been found than in typically developing (TD) children, possibly due to lifestyle factors such as unhealthy diet, being overweight or obese and less time spent outside (4, 5). However, the underlying associations of vitamin D3 deficiency in ASD are still poorly understood.

Two major forms of vitamin D exist, including vitamin D2 or ergocalciferol and vitamin D3 or cholecalciferol, which is more relevant in humans (4, 6, 8). Vitamin D3 plays a major role as prohormone and controls the expression of about 3% of our genes (3, 6). Chemically, vitamin D3 is a secosteroid that is strongly involved in the regulation of calcium levels. Synthesis of vitamin D3 in the skin from cholesterol following exposure to ultraviolet B (UVB) in sunlight is the major natural source (for 90%), and only a limited amount of vitamin D3 is derived from the diet or dietary supplement (9). To become activated, vitamin D must first be converted through hydroxylation in the liver into calcidiol, or 25-hydroxy vitamin D [25(OH)D] (4, 6, 8). Next calcidiol is converted in the kidneys into calcitriol or vitamin D [1,25 (OH)_2_D], the potent neurosteroid that helps control brain cell growth, and acts on vitamin D receptor molecules found in most brain cells (3, 10).

Recommendations for vitamin D3 serum levels vary. With a cut-off of serum 25(OH)D < 50 nmol/L, it is estimated that about 30% of adults have vitamin D3 deficiency and about 60% have insufficiency [serum 25(OH)D 50–75 nmol/L] (8). In contrast to older guidelines, the consensus is now that a minimum <75 nmol/L is a reasonable cut-off. Actually, the discussion in the field is more toward even higher cut-off criteria than lower (4, 6).

Vitamin D3 plays a major role in calcium and bone metabolism. Research over the last decade has demonstrated the diverse functions of vitamin D3 throughout the brain (3–7). For instance, vitamin D3 is involved in brain proliferation, differentiation, neurotrophism, neuroprotection, neurotransmission, myelination, and neuroplasticity (3, 4, 6, 7). As such, reduced levels of vitamin D3 may lead to suboptimal brain development and deficiency may result in increased risk of developing psychopathology. In adults, vitamin D3 deficiency is associated with depressive disorders, schizophrenia, Parkinson’s disease and Alzheimer’s disease (4, 7, 10).

Most studies in children and adolescents with psychiatric disorders analysing vitamin D status have investigated an association with ASD; while only a limited number of children with other psychiatric disorders have been studied with respect to vitamin D3 status so far. These have been assembled in a recent systematic review indicating that vitamin D is indeed associated with a broad area of mental health problems in children (11). For children with ASD, two recent meta-analyses demonstrated that children and adolescents with ASD had significantly lower vitamin D3 blood levels than TD children (4, 5). Moreover, it has been hypothesized that vitamin D3 deficiency may increase risk of ASD by altering brain development (3–7).

Additionally, lifestyle factors predisposing for reduced vitamin D3 levels such as poor diet quality, being overweight and reduced exposure to sunlight by an imbalance between indoor and outdoor activities may be more common in children with psychiatric disorders. In fact, multiple studies, mostly in adults, have reported associations between psychiatric disorders and unhealthy lifestyles (12). Children with ASD show higher-than-average rates of obesity and are often less active outdoors (13–16) which may have a negative effect on vitamin D3 status. A meta-analysis of vitamin D deficiency and obesity in adults and children in general population revealed inverse associations between BMI and vitamin D3 levels, regardless of age group (17). However, thus far, little is known about clinical associations between vitamin D3 and BMI in children with different psychiatric disorders.

Here, we aim to explore the prevalence of vitamin D3 in children with a psychiatric diagnosis including children with ASD or with internalizing disorders (mood and anxiety disorders). In addition, we will further investigate potential associations between vitamin D3 and effects of being overweight or obese in this population. We hypothesize that: (1) vitamin D3 deficiency is similarly present across diagnostic groups; and (2) BMI is inversely related to vitamin D3 levels in children with psychiatric disorders.

## Materials and methods

As part of ongoing explorative assessment on somatic comorbidity and lifestyle in children and adolescents with psychiatric disorders, we retrospectively reviewed documents and medical records between October 2020 and October 2021 including physical and laboratory measurements from patients diagnosed with ASD or internalizing disorders (mood and anxiety disorders) carried out between October 2017 and October 2020 at the outpatient clinic of Karakter University Centre for Child and Adolescent Psychiatry. Karakter UC provides psychiatric care for children and adolescents with an average or high intelligence level. In the Netherlands, referral to a University center, such as Karakter is only done if severe forms of psychopathology are present.

The authors assert that all procedures contributing to this work comply with the ethical standards of the relevant national and institutional committees on human experimentation and with the Helsinki Declaration of 1975, as revised in 2008. The local Medical ethics committee approved based on the principal of good clinical practice.

### Study sample

Study sample included children aged 6–18 with ASD (*n* = 54) and children with internalizing disorders (*n* = 39). Internalizing disorders included mood and anxiety disorders (i.e., depressive disorder, agoraphobia, generalized anxiety disorder, obsessive compulsive disorder, panic disorder, posttraumatic stress disorder, separation anxiety disorder, social phobia, and specific phobia). We included children for whom data on vitamin D3 status and BMI were available. Exclusion criteria were restrictive eating disorders with poor overall nutrition status and children who were supplemented with vitamin D3 at the time of assessment.

### Measures

#### Physical assessment

Physical examination included measurement of weight and height. The prevalence of children being underweight, overweight or obese was defined by the cut-off values of BMI references per age group according to the International Obesity Task Force (18). Ethnicity of the child was based on the parental country of birth. If both parents were non-Dutch, mother’s ethnicity determined the ethnicity of the child. Vitamin D3 levels were measured in a venous blood sample, which was processed at local laboratory facilities. We used the vitamin D3 reference values from the American Academy of Pediatrics Committee on Nutrition and The Institute of Medicine. Vitamin D3 insufficiency was defined as levels of 50–75 nmol/L; and deficiency as levels below <50 nmol/L (19–21).

#### Psychiatric assessment

During the first appointment a psychologist and child and adolescent psychiatrist interviewed the child and caregivers. Next, a mental state examination and physical examination was carried out by the child and adolescent psychiatrist or a nurse practitioner supervised by a child and adolescent psychiatrist. The psychiatric diagnosis was based on DSM-IV-TR (22) and/or DSM 5 criteria (23). Consensus diagnoses were established by a multidisciplinary team consisting of two child and adolescent psychiatrists, psychologists and two nurse practitioners.

### Data analysis

Baseline characteristics of patients were reported using frequency distributions for the categorical variables and mean ± standard deviation (±SD) for the continuous variables. We reported frequency distributions for Vitamin D3 deficiency (levels below <50 nmol/L) and BMI categories according the classification tables by TNO (International Obesity Task Force) (18) for the different diagnostic groups as well as the total group. A chi-square test was conducted between the outcome variables and primary diagnosis. Next, following tests for normal distribution, we ran univariate linear regressions separately for boys and girls and according to the different diagnostic groups to assess the effect of BMI on vitamin D3 levels. To assess linearity a scatterplot of BMI against vitamin D3 was plotted separately for boys and girls and according to the different diagnostic groups. A *p* value < 0.05 (two-sided) was considered statistically significant. All analyses were conducted using SPSS version 24 (IBM Corporation, Armonk, NY, United States).

## Results

**FIGURE 1:**
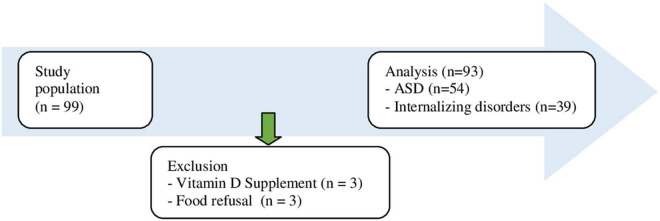
Flowchart of study inclusion.

Of the 99 patients, three patients were excluded because of eating disorders (anorexia nervosa with food refusal), and three because of supplementation with vitamin D3 at moment of laboratory assessment (Figure 1 flowchart).

Table 1 shows the baseline demographic and clinical characteristics of the participants. Of the 93 patients included, 47 were female (50.5%) with mean age of 14.4 years (range 6–18, SD 2.5). Over 50% of the children were 15–18 years. A total of 92.5% of the children were Dutch (Northern European descent). Psychiatric diagnoses were categorized in two main groups with ASD in 58.1% and internalizing disorders (mood and anxiety disorders) in 41.9% of the children as primary diagnoses. In total, 72 children were treated with psychiatric medication (77.4%) at moment of physical and laboratory assessment, comprising second generation antipsychotics (*n* = 53), antidepressants (*n* = 22), or stimulants (*n* = 27). Physical assessment displayed that 25.8% of the children were overweighed and 10.8% obese, according to BMI. Blood testing showed lower vitamin D3 deficiency (<50 nmol/L) in 77.4% (72/93) in the total group of children.

**TABLE 1:** Descriptive of the study population (*n* = 93).

		*n*	%
Age	6–11 year	8	8.6
	12–14 year	32	34.4
	15–18 year	53	57.0
Sex	Female	47	50.5
	Male	46	49.5
Dutch	Yes	86	92.5
	No	7	7.5
Primary psychiatric diagnosis	ASD	54	58.1
	ID	39	41.9
Psychiatric medication	Yes	72	77.4
	No	21	22.6
BMI^1^	(Severe) underweight	2	2.2
	Normal	57	61.3
	Overweight	24	25.8
	Severe overweight (obesitas)	10	10.8
Vitamin D3	<50 nmol/L	72	77.4
	≥50 nmol/L	21	22.6
		
		**Mean**	**SD**
		
Length (cm)		167.2	15.0
Weight (kg)		62.1	18.4
BMI (kg/m^2^)		21.9	4.6
Age (years)		14.4	2.5
Vitamin D3 (nmol/L)		40.9	16.6

BMI, body mass index; ASD, autism spectrum disorder; ID, internalizing disorders (depression and anxiety); SD, standard deviation. ^1^Underweight, overweight and obesity prevalence for the children, were defined by the cut-off values on BMI references and age according to the International Obesity Task Force (18).

Table 2 presents BMI outcome, vitamin D3, sex and psychiatric medication separately for children with ASD and children with internalizing disorders. There were significantly more boys with ASD (72.2%) than boys with internalizing disorders (17.9%). Children with a primary diagnoses of an internalizing disorder more often had vitamin D3 deficiency (79.5%) compared to children with ASD (75.9%), however, these differences were not significant. Furthermore, 14.8% of children with ASD were classified as having severe overweight, whereas 5.1% of the children with ID were classified as having severe overweight. However, these differences were also not significant.

**TABLE 2:** Body mass index outcome, Vitamin D3, sex and psychiatric medication for children with ASD and children with internalizing disorders.

		Primary psychiatric diagnosis				*p*-Value^2^
		ASD (*n* = 54)		ID (*n* = 39)		
		*n*	%	*n*	%	
Sex	Female	15	27.8	32	82.1	0.000
	Male	39	72.2	7	17.9	
Vitamin D3	<50 nmol/L	41	75.9	31	79.5	0.685
	≥50 nmol/L	13	24.1	8	20.5	
Psychiatric medication	No	16	29.6	5	12.8	0.056
	Yes	38	70.4	34	87.2	
BMI^1^	(Severe) underweight	1	1.9	1	2.6	0.519
	Normal	32	59.3	25	64.1	
	Overweight	13	24.1	11	28.2	
	Severe overweight (obesitas)	8	14.8	2	5.1	

BMI, body mass index; ASD, autism spectrum disorder; ID, internalizing disorders. ^1^Underweight, overweight, and obesity prevalence for the children, were defined by the cut-off values on BMI references and age according to the International Obesity Task Force (18). ^2^Chi-square tests.

A multiple regression analysis was run to predict vitamin D3 levels from BMI and age in the total group and to determine how much of the variation in the dependent variable (vitamin D3 level) could be explained by the independent variables (BMI and age). As presented in Table 3, in the total group in model 1, higher BMI levels predicted lower vitamin D3 scores significantly (*p* < 0.016). In model 2, the multiple regression model including age, significantly predicted vitamin D3, F(2, 90) = 5.888, *p* < 0.004, adj. *R*^2^ = 0.096. Age was the only factor contributing significantly to the prediction (*p* = 0.022).

**TABLE 3:** Multiple regression results for Vitamin D3 level for boys and girls separately.

		B	SE B	ß	*F*	*R* ^2^	Adj. *R*^2^
Total group							
Model 1					6.054*	0.062	0.052
	(Constant)	60.56	8.16				
	BMI	−0.90*	0.37	−0.25			
Model 2					5.888*	0.116	0.096
	(Constant)	78.17	10.98				
	BMI	−0.65	0.37	−0.18			
	Age	−1.60*	0.690	−0.24			
Boys							
Model 1					4.995*	0.102	0.082
	(Constant)	66.69	11.88				
	BMI	−1.19*	0.53	−0.32			
Model 2					4.410*	0.170	0.132
	(Constant)	86.17	15.52				
	BMI	−0.91	0.54	−0.24			
	Age	−1.81	0.96	−0.27			
Girls							
Model 1					1.376	0.030	0.008
	(Constant)	54.15	11.35				
	BMI	−0.60	0.51	−0.17			
Model 2					1.628	0.229	0.101
	(Constant)	69.78	16.07				
	BMI	−0.38	0.53	−0.11			
	Age	−1.39	1.02	−0.21			

ASD, autism spectrum disorder; BMI, body mass index; Model, “Enter” method in SPSS Statistics; B, unstandardized regression coefficients; SE B, standard error of the coefficient; ß, standardized coefficients; *R*^2^, coefficient of determination; Adj. *R*^2^, adjusted *R*^2^. *p < 0.05.

**FIGURE 2:**
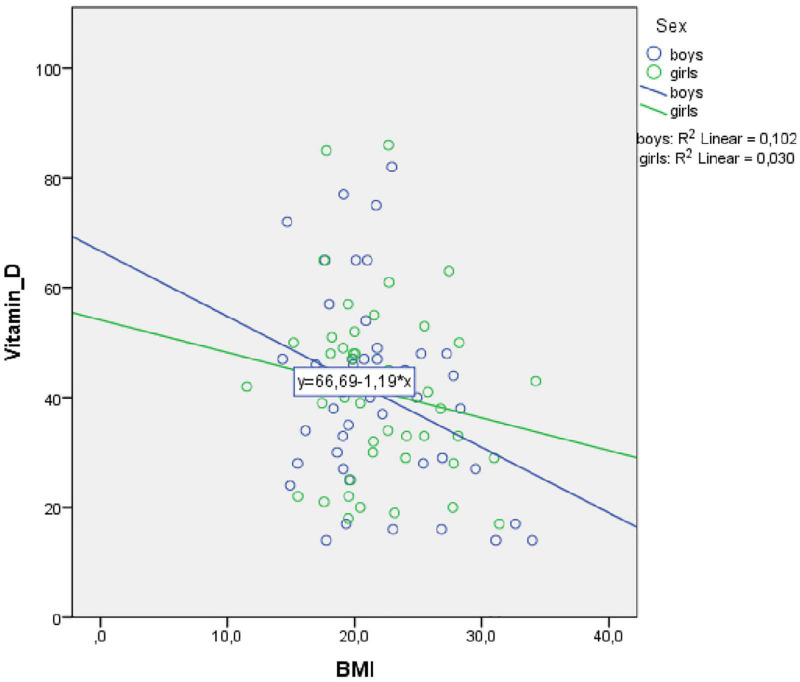
Scatterplot of vitamin D3 body mass index (BMI) for girls and boys separately.

Because our sample included more boys with ASD than internalizing disorders, the role of vitamin D3 and BMI stratified for sex was further explored. For boys, the prediction equation was: Vitamin D3 = 86.2–0.9 × BMI − 1.8 × AGE. BMI levels predicted vitamin D3, F(2, 43) = 4.410, *p* = 0.018, accounting for 17.0% of the variation in vitamin D3 with an adjusted *R*^2^ = 13.0%, a small effect size according to Cohen (24). For girls, the prediction equation was: Vitamin D3 = 69.8 − 0.4 × BMI − 1.3 × AGE. BMI levels, however, did not significantly predict vitamin D3 levels, F(2, 44) = 1.63, *p* = 0.205. Regression coefficients and standard errors can be found in Table 3. The regression slopes for boys and girls are graphically presented in Figure 2.

**FIGURE 3:**
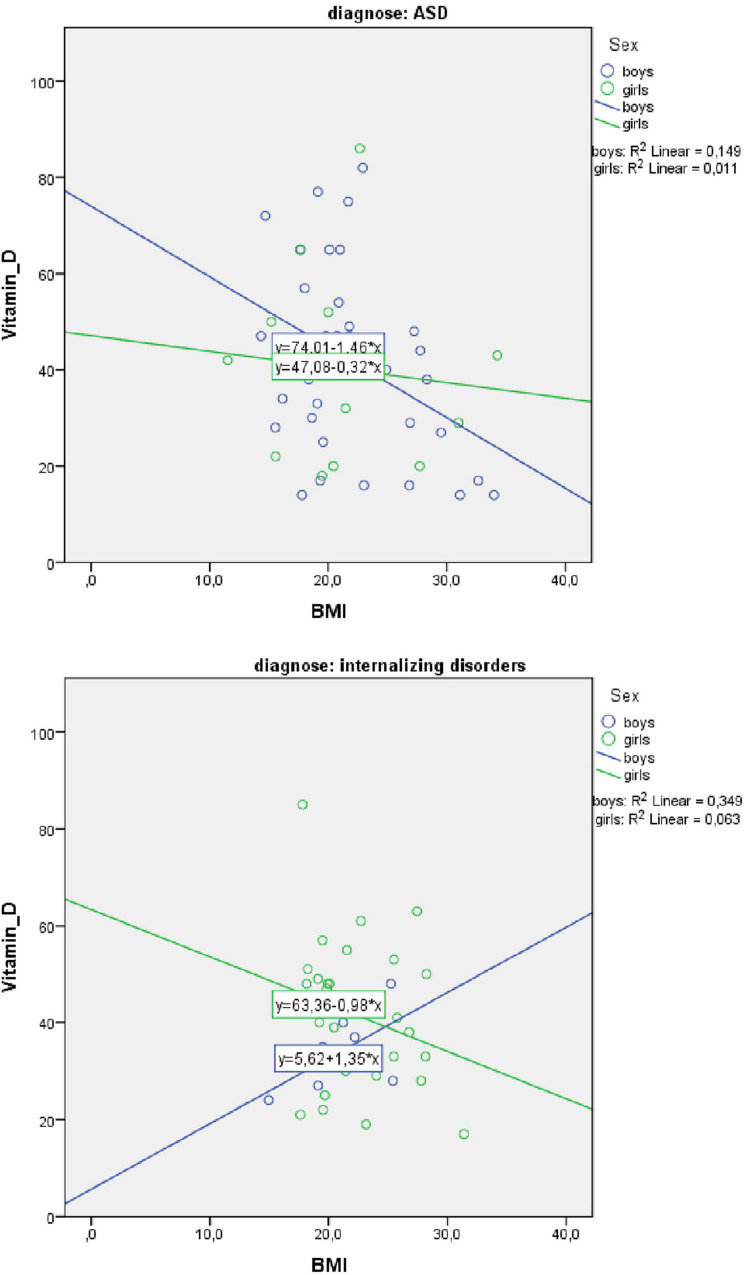
Scatterplot of body mass index (BMI) and Vitamin D3 level for boys and girls separately for autism spectrum disorder (ASD) and internalizing disorders.

Also, the primary diagnosis was stratified, due to the higher percentages of children with ASD and obesity (Table 4 and Figure 3). For boys with ASD, higher BMI scores partially predicted lower vitamin D3 levels: vitamin D3 = 94.3–1.2 × BMI − 1.9 × AGE. BMI and age predicted significantly vitamin D3, F(2, 36) = 5.13, *p* = 0.011 accounting for 22.2% of the variation in Vitamin D3 with an adjusted *R*^2^ = 17.9%. For the other models, the variables did not add significantly to the prediction.

**TABLE 4:** Results of a linear regression analysis of BMI and Vitamin D3 level for boys and girls and for ASD and internalizing disorders separately.

ASD								Internalizing disorders							
		B	SE B	ß	*F*	*R* ^2^	Adj. R^2^			B	SE B	ß	*F*	*R* ^2^	Adj. *R*^2^
Boys								Boys							
Model 1					6.494*	0.149	0.126	Model 1					2.68	0.349	0.219
	(Constant)	74.02	12.91						(Constant)	5.617	17.65				
	BMI	−1.47*	0.58	−0.39					BMI	1.353	0.83	0.59			
Model 2					5.131*	0.222	0.179	Model 2					1.11	0.357	0.035
	(Constant)	94.29	16.71						(Constant)	1.398	27.26				
	BMI	−1.18*	0.58	−0.31					BMI	1.216	1.1	0.53			
	Age	−1.89	1.03	−0.28					Age	0.473	2.12	0.11			

**ASD**								**Internalizing disorders**							

Girls								Girls							
Model 1					0.146	0.011	−0.065	Model 1					2.001	0.063	0.031
	(Constant)	47.08	18.49						(Constant)	63.36	15.66				
	BMI	−0.33	0.85	−0.11					BMI	−0.98	0.69	−0.25			
Model 2					1.784	0.229	0.101	Model 2					1.051	0.068	0.003
	(Constant)	71.19	21.45						(Constant)	73.52	30.14				
	BMI	0.49	0.9	0.16					BMI	−0.99	0.7	−0.254			
	Age	−3.04	1.65	−0.54					Age	−0.65	1.64	−0.071			

ASD, autism spectrum disorder; BMI, body mass index; Model, “Enter” method in SPSS Statistics; B, unstandardized regression coefficients; SE B, standard error of the coefficient; ß, standardized coefficients; *R*^2^, coefficient of determination; Adj. *R*^2^, adjusted *R*^2^. *p < 0.05.

## Discussion

In this study high rates of vitamin D3 deficiency were found in children with psychiatric disorders, predominantly ASD and internalizing disorders. Additionally, the results showed that BMI was inversely related to vitamin D3 in the total group of children and more specifically in boys with ASD. Our findings overall indicate that children with a variety of psychiatric disorders are at risk of vitamin D3 deficiency, especially in co-occurrence with overweight and obesity.

Vitamin D3 deficiency is associated with a range of adverse somatic and psychiatric outcomes (cardiovascular, diabetes, depression, neurobiological disorders, neurodegenerative diseases, cancer, and psychosis) and is of major interest, considering its role in fundamentally biological processes (9, 24). Previous studies on the role of vitamin D3 in the pathogenesis of ASD in childhood have been performed, however, specific mechanisms are still unclear (3–5). In our study high percentages of low vitamin D3 levels were not specific for children with ASD but also observed in children with internalizing disorders. The results of this study showed a prevalence of vitamin D3 deficiency (<50 nmol/L) in the total group of children with severe psychiatric disorders of 77%; in ASD 76% and in internalizing disorders 80%. This is much higher compared to a study in the general population of Dutch children where a prevalence of 30% was previously described (25). However, studies vary in reports of vitamin D levels in general population worldwide (26).

Prevalence of vitamin D3 deficiency (<50 nmol/L) in a study of 307 adolescents (11–18 year) in the United States was 42% (27). In a study in the United Kingdom (*n* = 1102), 35% of the 4–8 year old children were vitamin D deficient (<50 nmol/L) (28). In Beijing China, a prevalence of vitamin D (<50 nmol/L) 57.8% was found (29). The Canadian Health Measures Survey reported a prevalence of vitamin D insufficiency (<50 nmol/L) of 13% in children aged 6–11 years from April to October (30). In Germany in 10.015 children (1–17 years) prevalence of vitamin D < 30 nmol/L was 12.5% for boys and girls; and vitamin D 30 to <50 nmol/L was 32.7% in boys and 33.5% in girls (31). A general population study of 1,829 adolescents in India reported a high prevalence of vitamin D deficiency (<50 nmol/L) of 97% (32). Also, within our study population, we observed that older children had a higher risk of vitamin D deficiency. Although infants are at risk for disease such as vitamin D-associated rickets, previous studies in higher-resource countries reported lower vitamin D levels in adolescents compared to toddlers (26, 31). Different, possibly combined explanations are possible; such as less supplementation during adolescence and or an imbalance between in- and outdoor activities (in favor of indoor activities) (25, 28).

The variations in vitamin D level among studies could be attributed to difference in study design, inclusion of different age groups, definition of insufficiency or deficiency, and geographical differences (26). Populations at increased risk for vitamin D deficiency include immigrant/refugee children moving to higher-latitude countries (33, 34), dark skin color or skin pigmentation (25), children with chronic diseases that decreases fat absorption (35), children receiving anti-epileptic medications (36), and obese children (37). According to our study, children with psychiatric disorders also appear to be at high risk for vitamin D3 deficiency.

Moreover, high levels of obesity were found in children with ASD and internalizing disorders. As obesity is an important risk factor for cardiovascular disease and increased morbidity rates during later life, it is important to be aware off the risk factors for obesity, such as some types of medication, the psychiatric disorder itself, genetic variations associated with obesity and last but not least lifestyle related factors (e.g., disordered sleep, insufficient physical activity, diet and eating behavior) (38, 39). A significant inverse association between vitamin D level and BMI in this study is in line with studies in typically developing children (17, 26). The underlying mechanisms associating low vitamin D in obesity include volumetric dilution, sequestration into adipose tissue, limited sunlight exposure, and decreased vitamin D synthesis in the adipose tissue and liver (40). For example, it is speculated that adipose tissue is responsible for lower circulating 25(OH)D levels due to a greater pool of distribution for the fat soluble vitamin (4, 40, 41).

Interestingly, in the present study, vitamin D3 deficiency was particularly common in boys with ASD with higher BMI. It could be hypothesized that life style factors (unhealthy diet and limited physical activity outdoors) may contribute to the association between high BMI and low vitamin D3 levels in boys with ASD more than in girls, but other mechanisms may play a role. For instance, sexual hormones, estrogen and testosterone, appear to have different effects on vitamin D metabolism, which may explain sex differences of vitamin D status (3, 42–44). However, further studies need to explore sex specific differences in vitamin D3 levels and obesity.

There are several limitations regarding this study. First, this study was performed to investigate whether lower levels of vitamin D3 are predominantly associated with ASD, or are related to a broad spectrum of psychopathology in a broader sense, e.g., across disorders. While the latter appeared to be the case, no conclusions can be drawn about more average or less severe psychopathology. Another limitation is that, although we have included children with a variety of internalizing child psychiatric diseases at different ages, the diagnostic subgroups were relatively small and did not allow further analysis. Also, there could be an overlap between children with ASD and internalizing problems (anxiety or mood symptoms). However, children diagnosed with internalizing disorders did not have a neurodevelopmental disorder (e.g., ASD). Our main focus was to explore if vitamin D3 is associated with a broad area of psychiatric disorders in children. It would be interesting for future research to investigate whether vitamin D3 deficiency is more present in children with ASD in combination with internalizing symptoms. A large cohort in ASD children in which two groups of internalizing and externalizing symptoms are dichotomized is preferably. Third, we did not include a control group with typically developing children to compare vitamin D3 levels. Fourth, the cross-sectional and exploratory design does not allow further exploration of intricate relationships between BMI and vitamin D3 levels in time, which clearly will be an important topic to address in future studies. Unfortunately, the study was underpowered. We calculated the achieved power in a *post hoc* analysis for the main linear multiple regression in boys with ASD. With a α of 0.05, a sample size of 39 (boys with ASD), two predictors and a partial *R*^2^ of 0.149, the achieved power was 0.60. We therefore advice to increase the population size in future studies in order to replicate the results. Furthermore we did not have sufficient data to explore relation with different ethnicity, socio-economic status and to determine seasonal variations in serum vitamin D3 levels and effects on mental health.

Future research into vitamin D3 in child and adolescent psychiatric populations should take the following recommendations into account:

1) The role of vitamin D3 in psychiatric and somatic diseases needs further exploration, for instance how it relates to different factors (such as BMI, age, sex, diet, ethnicity, season, etc.), but also what the effects of supplementation are in psychiatric diagnoses. With respect to vitamin D3 supplementation many questions remain present. What is the optimal dosage for supplementation and how should vitamin D3 be administered? To what extent are follow up measures of vitamin D3 status necessary? How long should supplementation be provided?
2) A trans-diagnostic approach, including younger children and children with intellectual disability may provide additional insights. Ideally, larger diagnostic groups should be included allowing a higher degree of statistical evidence.
3) A longitudinal study design is preferable, which allows insights in developmental trajectories related to vitamin D3 status, diagnostic status, effects of sex differences and BMI.
4) At a broader public health level, further research is required into the effectiveness of interventions that promote a healthy lifestyle, especially in child psychiatric populations.

In conclusion, this study demonstrated high rates of vitamin D3 deficiency in children with psychiatric disorders, and an inversely relation between BMI and vitamin D3 in the total group of children, most pronounced in older boys with ASD. Vitamin D3 deficiency is common in children and adolescents with psychiatric disorders and it is vital to increase clinicians’ awareness of this common and remediable risk factor. Identifying the specific role of vitamin D3 in psychiatric and somatic comorbidities may potentially provide better insights in disease etiology and may have important implications for interventions and future research. Addressing lifestyle factors such as diet and time spent outdoors may improve physical and mental health in children and adolescents with psychiatric disorders.

## Data availability statement

The raw data supporting the conclusions of this article will be made available by the authors, without undue reservation.

## Ethics statement

The studies involving human participants were reviewed and approved by Radboudumc. Written informed consent to participate in this study was provided by the participants’ legal guardian/next of kin.

## Author contributions

JM conceived and developed an initial draft manuscript in consultation with JZ, MD-B, and WS who regularly provided extensive feedback. HK and JM carried out the analyses and interpretation. All authors contributed to the final version of the manuscript.

## Abbreviations

25(OH)D: 25-hydroxyvitamin D
ASD: autism spectrum disorder
Calcitriol: 1,25(OH)D2
BMI: body mass index.
